# Supercritical Antisolvent Fractionation of Antioxidant Compounds from *Salvia officinalis*

**DOI:** 10.3390/ijms22179351

**Published:** 2021-08-28

**Authors:** Raquel Mur, Juan I. Pardo, M. Rosa Pino-Otín, José S. Urieta, Ana M. Mainar

**Affiliations:** 1 GATHERS Group, Aragón Institute of Engineering Research (I3A), Universidad de Zaragoza, C/Mariano Esquillor s/n, 50018 Zaragoza, Spain; 649396@unizar.es (R.M.); jupardo@unizar.es (J.I.P.); urieta@unizar.es (J.S.U.); 2 Campus Universitario Villanueva de Gállego, Universidad San Jorge, Autovía A-23 Zaragoza-Huesca Km. 299, 50830 Villanueva de Gállego, Spain; rpino@usj.es

**Keywords:** advanced separation processes, supercritical antisolvent fractionation, rosmarinic acid, caffeic acid, chlorogenic acid, antioxidant activity, QSAR

## Abstract

The increasing interest towards greener antioxidants obtained via natural sources and more sustainable processes encourages the development of new theoretical and experimental methods in the field of those compounds. Two advanced separation methods using supercritical CO_2_ are applied to obtain valuable antioxidants from *Salvia officinalis*, and a first approximation to a QSAR model relating molecular structure with antioxidant activity is explored in order to be used, in the future, as a guide for the preselection of compounds of interest in these processes. Separation experiments through antisolvent fractionation with supercritical CO_2_ were designed using a Response Surface Methodology to study the effect of pressure and CO_2_ flow rate on both mass yields and capability to obtain fractions enriched in three antioxidant compounds: chlorogenic acid, caffeic acid and rosmarinic acid which were tracked using HPLC PDA. Rosmarinic acid was completely retained in the precipitation vessel while chlorogenic and caffeic acids, though distributed between the two separated fractions, had a major presence in the precipitation vessel too. The conditions predicted for an optimal overall yield and enrichment were 148 bar and 10 g/min. Although a training dataset including much more compounds than those now considered can be recommended, descriptors calculated from the σ-profiles provided by COSMO-RS model seem to be adequate for estimating the antioxidant activity of pure compounds through QSAR.

## 1. Introduction

The importance of antioxidants coming from natural sources as bioactive compounds, and their interest in the pharmaceutical, food and cosmetic industries, is increasingly [1,2,3] recognized worldwide. Moreover, society and government agencies progressively demand not only for safer products for humans and the environment, but also that these products should be obtained through clean, non-polluting procedures. Over many years, the extraction of bioactive compounds from natural sources and their subsequent fractionation or isolation have been conducted by means of conventional methods, many of which are based in the use of organic solvents that can be harmful for the environment and human health. Then, the development of more sustainable processes, as free as possible of organic solvents, is of great importance. For this reason, supercritical fluid, especially supercritical carbon dioxide (sc-CO_2_), technology has gained importance and is widely used for extraction, fractionation and isolation of bioactive compounds from plants [4] or animal parts [5]. CO_2_ is non-flammable, non-toxic, available at low cost with high purity and its critical pressure and temperature (P_c_ = 74 bar T_c_ = 31 °C) are moderate [4]. This last circumstance makes it very suitable for the extraction of thermolabile compounds. Besides, sc-CO_2_ has zero surface tension, low viscosity and its diffusivity is two or three times higher than that of other fluids. CO_2_ exhibits a polarity similar to pentane, and for this reason is suitable for the extraction of lipophilic substances [6]. Although, CO_2_ has the disadvantage that it lacks the polarity required to extract polar substances, this shortcoming could be surmounted by using a co-solvent such as a short chain alcohol [4]. As a result of its properties, sc-CO_2_ leads to effective and quick extractions and also eliminates clean-up steps because it can be easily and completely removed by lowering the pressure in which case it becomes a gas.

Supercritical carbon dioxide technology allows working in several different modes. By far the most used and studied is supercritical fluid extraction (SFE). More than 90 SFE processes are now performed by this technique. It has been applied to plants because they are sources of a great number of bioactive substances such as hydrocarbons (monoterpenes and sesquiterpenes) and oxygenated compounds (aldehydes, acids, alcohols, ketones, phenols, etc.) [7]. Most studies on SFE include investigations of the effect on the yield of extraction owing to parameters such as temperature, pressure, size of sample, modifiers (co-solvent), flow rate of fluid, and sample moisture [4,5,6,7,8,9,10]. Our group has previously studied the extraction of *Hyssopus officinalis* [11], *Salvia officinalis* [12], *Artemisia absinthium* [13], *Persea indica* [14], *Lippia alba* [15], *Citrus aurantium amara* [16]. Regarding the extraction of *Salvia officinalis*, in the last 10 years, several papers have been published with variations in both the origin of the plant and the conditions used for its extraction, which mean different results in the yield and composition of the extracts [17,18,19,20,21,22].

Another interesting and less studied technique is the supercritical antisolvent fractionation (SAF) [4], in which the non-polarity of CO_2_ can be used to precipitate some compounds from a solution into solid particles, whose shape and diameter could be controlled according to the operational conditions [4,23], while the rest remain dissolved. That is, two fractions can be obtained that are enriched in different components. Our group has already performed investigations in this technology aiming to obtain fractions enriched in bioactive compounds: *Persea indica* [24], grape seeds [25], *Artemisia absinthium* [26] and *Lavandula luisieri* [23]. There are no SAF studies for *Salvia officinalis*, although Ganan et al. [27] performed a fractionation of its essential oil using different technique.

In relation to the SAF process, it exhibits conceptual similarities with the supercritical antisolvent micronization (SAS) [4,28], a process widely used in the pharmaceutical industry to obtain small particles of some drugs. Complex mathematical models [29,30] have been also developed to understand particularities of SAS such as jet hydrodynamics, mass transfer and phase equilibrium data as well as the buoyancy effect on the fluid. Besides, the technique of coprecipitation with polymers in sc-CO_2_ has also been applied to stabilize antioxidants in order to preserve them and avoid their degradation [31,32,33,34].

Now, this work focusses on the application of SAF to extract *Salvia officinalis* L., commonly known as sage. *Salvia officinalis* belongs to the *Salvia* genus, which includes over 900 species [35]. It is native to Middle East and Mediterranean areas. In the traditional medicine of Europe, it has been used to treat some different illnesses, such as dyspepsia or inflammations in the throat and skin [36]. In recent years, many researches have revealed a wide range of pharmacological activities including antioxidant [37,38], antimicrobial [39,40], anticancer [41,42], anti-inflammatory [43], antimutagenic [37], cognitive and memory-enhancing [38,44]. However, one of the major uses of *Salvia officinalis* extracts and essential oil lies in the cosmetic and perfume industries. In fact, those extracts are so important that they are controlled under various regulations (Directive 2004/24/EC, CFR Title 21 from FDA, etc.) and have their own CAS (Chemical Abstracts Service) registry numbers, 8022-56-8/84082-79-1, and EINEC number (European Inventory of Existing Commercial chemical Substances): 282-025-9/282-025-9 [45]. According to the EU cosmetic ingredient database, CosIng, the extracts of this plant have several functions as a cosmetic: anti-seborrheic, antimicrobial, antioxidant, astringent, cleansing, deodorant, skin conditioning, skin protecting, soothing and tonic [46].

Due to the beneficial properties of its extracts, their composition has been studied in detail [20,41,47] and SFE of *S. officinalis* has been also investigated [48,49]. As could be expected, the composition and resulting properties of the extracts are highly dependent on extraction conditions apart from other factors such as the origin of the plant or its chemotype [50,51]. As it is well known, extraction depends on solution and diffusion processes which are governed by thermodynamic laws corresponding to phase equilibria, and kinetic parameters associated with transport phenomena, respectively [52]. Over the years, different thermodynamic models have been proposed in order to correlate or predict solubilities in supercritical fluids such as those based on equations of state [53] or regular solution theory [54]. Additionally, more complex models, including the integration of differential mass balances, can be found—models that interpret the extraction curves giving mass yields against extraction time [4,28].

In this work, the influence of pressure (80–160 bar) and CO_2_ flow rate (10–60 g/min) in a SAF process on the yields for each fraction as well as on the enrichment (fractionation) of three compounds, namely, rosmarinic acid (RA) [55,56,57], caffeic acid (CAF) [58] and chlorogenic acid (CHA) [59] have been studied. These phenolic acids were chosen because they exhibit a significant antioxidant activity [60,61]. A response surface methodology (RMS) based on central composite design (CCD) was used to carry out the experimental design. It must be said that this SAF process is intended as a stage of a global sustainable process in which SFE would be the first applied to defat the plant material which would be afterwards subjected to maceration with ethanol to obtain the polar and, to us, more interesting compounds that would, finally, undergo the SAF process.

It must be also considered that a lot of experimental and theoretical studies are necessary to reach a sufficiently satisfactory level of knowledge in the field of separation of bioactive compounds. On the one hand, due to the wide variety of raw materials susceptible to be treated and their composition variability (chemotype, farmland, harvest, climatology) and to the large series of compounds that can be obtained, this type of separation processes is far from being exhaustively studied, though several industrial applications are already in operation. On the other hand, together with the already indicated operational models, another types of approximations are necessary when a progressive line of scaling-up from laboratory to pilot and industrial scales is foreseeable as a result of the interest in and consequent demand for the obtained products. Another relevant facet is the assessment of the quality of the products. In this respect, because of the growing attention paid to obtaining high added value products, such as antioxidants, semiempirical models that would allow the discrimination of the most active compounds are of great interest as these models can be used to focus processes such as SAF in the most promising bioactive compounds. This has led us to explore the possibility of attempting the development of a Quantitative Structure Activity Relationship (QSAR) model by using molecular descriptors derived from the σ-profiles of antioxidant compounds provided by the Conductor-like Screening Model for Real Solvents (COSMO-RS model). COSMO-RS model, first proposed by Klamt [62] and afterwards refined in [63], is a continuum solvation model that combines quantum chemical theory and statistical thermodynamics. From optimized three-dimensional structures of the molecules COSMO-RS generates a 3-D distribution of surface polarization charge-densities, σ, of the compounds from which the corresponding 2-D σ-profiles are obtained. σ-profiles are histograms that provide the relative amount of molecular surface with a given polarization charge-density σ. The model was originally intended to calculate solvent properties, vapor-liquid or liquid-liquid equilibrium phase diagrams, vapor pressures or activity coefficients. From activity coefficients properties of real mixtures could be predicted. However, besides this original aim, it has also proven to be a useful tool to establish QSPR (Quantitative Structure Property Relationship) models for physical properties such as density [64,65], viscosity [65,66,67], electrical conductivity [68] and polarity [69] of different compounds or mixtures as well as adsorption phenomena [70,71]. Furthermore, QSAR models based on COSMO-RS have been developed for several bioactivity features, namely, cytotoxicity on leukaemia rat cell line [72], enzymes performance [73] and antimicrobial activity of ionic liquids [74]. However, as far as we know, the model that is intended to be studied in this work has not been used in the prediction of antioxidant activities as a guide before the selection of compounds of interest. Additionally, we take advantage of the experience of our research group in the use of the COSMO-RS model to predict vapor-liquid equilibria [75], excess molar enthalpies [76,77,78] and excess molar heat capacities [79,80,81,82] of binary liquid mixtures in order to advance towards the development of a QSAR model for antioxidant activity of terpenes and terpenoids.

## 2. Results and Discussion

### 2.1. Extraction Yields

In previous works, pre-treated plant material was submitted to a hexane maceration in order to eliminate non-polar compounds such as cuticular waxes before obtaining the polar and active compounds [23,26]. In this work this step was substituted by a green process with sc-CO_2_ because it is harmless and generates a final product without traces of residual solvent as CO_2_ becomes a gas at low pressures. The yield for the supercritical extraction was calculated using the Equation (1):

(1) $$Y_{SCE}\left(\mathrm{wt}.\%\right)=\left(\frac{mass\;{\left(g\right)}_{C1}+mass\;{\left(g\right)}_{C2}}{mass\;{\left(g\right)}_{\mathrm{plant}\;\mathrm{material}}}\right)\cdot 100$$

where mass(*g*)_C1_ and mass(*g*)_C2_ were the masses (grams) of extract collected in C1 and C2, respectively, and mass(*g*)_plant material_ was the initial mass (grams) of plant material loaded in the extractor. The extraction yield of the supercritical extraction, *Y*_SCE_, was 4.9%. Comparable results were obtained by S. A. Aleksovski et al. [83] (2.4–4.8%) and A. Dapkevicius et al. [84] (5.02%) at SFE conditions similar to those used in this work.

After the supercritical extraction the plant material was macerated in ethanol in order to obtain polar bioactive compounds. This solvent was used because of its good properties: it is nontoxic, biodegradable and has a high extractive capacity [23,85]. The yield of the maceration was calculated using Equation (2):

(2) $$Y_{M}\left(\mathrm{wt}.\%\right)=\left(\frac{{\mathrm{mass}}_{plant\;extract}\;\left(g\right)}{{\mathrm{mass}}_{plant\;material\;}\left(g\right)\;}\right)\cdot 100$$

where mass_plant extract_*(g)* was the mass (grams) of the solvent-free extract and mass_plant material_*(g)* was the initial mass (grams) of plant material subjected to maceration. The extraction yield obtained, Y_EtOH_, was 10.9%. This extract was redissolved again in ethanol to obtain the feed solution (FS) (3% wt. of extract) for the SAF process.

### 2.2. Supercritical Antisolvent Fractionation (SAF) Processes

Mass recovery yields for precipitation vessel (PV) and downstream vessel (DV) fractions, *Y*_PV_ and *Y*_DV_, were calculated using Equation (3):

(3) $$Y_{i}\left(\mathrm{wt}.\%\right)=\left(\frac{{\mathrm{mass}\;\mathrm{fraction}\;\mathrm{collected}}_{i}}{\mathrm{mass}\;\mathrm{of}\;\mathrm{FS}}\right)\cdot 100$$

where *i* was the collecting vessel: PV or DV. The overall recovery yield of the process, *Y*_SAF_, was defined according Equation (4):

(4) $$Y_{\mathrm{SAF}}\left(\mathrm{wt}.\%\right)=Y_{\mathrm{PV}}\left(\mathrm{wt}.\%\right)+Y_{\mathrm{DV}}\left(\mathrm{wt}.\%\right)$$

The concentration of a single compound in each sample was calculated using Equation (5):

(5) $$C_{i/j}\left(\mathrm{wt}.\%\right)=\left(\frac{mass\;of\;compound\;i\;in\;fraction\;j\left(g\right)}{mass\;of\;fraction\;j}\right)\cdot 100$$

where *i* means the compound analysed (RA, CHA, CAF) and *j* the fraction (FS, PV or DV) where the compound has been collected from. Once the concentrations were obtained, the Enrichment ratio *E*_i/j_ was calculated for each compound according to Equation (6):

(6) $$E_{i/j}=\frac{C_{i/j}}{C_{i/FS}}$$

where *j* is PV or DV. With these values a Relative Enrichment ratio $RE_{i}$ can be calculated using Equation (7):

(7) $$RE_{i}=\frac{E_{i/PV}}{E_{i/DV}}$$

The operational parameters to be set in a SAF process are pressure and temperature in PV and DV as well as the flow rates of CO_2_ and FS. Temperatures in PV and DV, pressure in DV and FS flow rate are fixed according to previous experiences in such a way that along with pressure in PV and CO_2_ flow rate supercritical conditions of the (CO_2_-ethanol) mixture would be ensured [25]. To statistically evaluate and optimize the influence of the variable operational parameters, namely, pressure in PV and CO_2_ flow rate, on yields and enrichment ratios a response surface methodology (RMS) based on central composite design (CCD) was employed. The values for pressure, *X*_P_*,* and CO_2_ flow rate, *X*_*Q*_CO_2___, were set between 80 and 160 bar and 10 and 60 g/min, respectively. In Table 1 the working conditions for every run can be found.

### 2.3. SAF Yields Statistical Analysis

The mathematical model for a two variable CCD allows correlating a dependent variable, *Y*, with some independent variables, *X_i_* and *X_j_*, through the following Equation (8):

(8) $$Y=\beta_{0}+\overset{2}{\underset{i=1}{\sum }}\beta_{i}X_{i}+\overset{2}{\underset{i=1}{\sum }}\beta_{ii}X_{i}^{2}+\overset{2}{\underset{i\neq j=1}{\sum }}\beta_{ij}X_{i}X_{j}$$

where *β*_0_ is the constant coefficient, *β*_1_ and *β*_2_ are linear coefficients, *β*_11_ and *β*_22_ are quadratic coefficients and *β*_12_ is an interaction coefficient. In this work, as said above, yields and enrichment ratios of bioactive compounds (RA, CAF, CHA) are the dependent variables while pressure and CO_2_ flow rate (encoded as *X*_P_ and *X*_*Q*_CO_2___, respectively) are the independent variables whose influence is under study.

In all the experiments a light-green powder precipitated in precipitation vessel (PV) and a green solution was obtained in downstream vessel (DV). Experimental values for PV (*Y*_PV_), DV (*Y*_DV_) and overall (*Y*_SAF_) yields are also shown in Table 1, where, as can be observed, *Y*_PV_ was always higher than *Y*_DV_, independent of the pressure and CO_2_ flow conditions. *Y*_PV_ oscillated between 53.0% (run 3; 92 bar-53 g/min) and 64.8% (run 2; 92 bar-17 g/min and run 6; 120 bar-35 g/min) whereas *Y*_DV_ changed between 7.1% (run 1; 80 bar-35 g/min) and 28.5% (run 11; 148 bar-17 g/min). *Y*_SAF_ varied from 69.0% to 85.1% (runs 1 and 11 respectively). It can be seen that PV yields are from 2.0 (run 4) to 8.7 (run 1) times the DV yields. A full recovery of the entire mass of solutes contained in the feed solution is not possible due to the dragging of the most volatile components through the vent valve [86] and the deposition of materials in dead spaces.

Only *Y_DV_* and *Y_SAF_* were successfully fitted to the mathematical model and used to determine the coefficients of Equation (8) that can be found in Table 2 along with the corresponding level of significance, *p*, the coefficient of determination, R^2^, and the standard deviation, s. According to the statistical analysis, *Y_DV_* depends on all the terms, although only the pressure (*β*_1_), the quadratic term of pressure (*β*_11_) and the CO_2_ flow rate (*β*_2_) are statistically significant (*p* < 0.05). Instead, *Y*_SAF_%, only depends on the terms of pressure (*β*_1_), CO_2_ flow rate (*β*_2_) and the quadratic term of pressure (*β*_11_), being all of them statistically significant.

**Figure 1:**
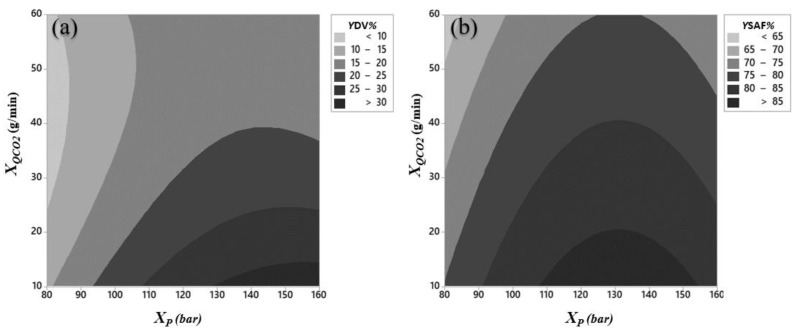
Contour plots of the yields: (**a**) at DV, *Y*_DV_% and (**b**) overall yield, *Y*_SAF_% as function of pressure, *X*_P_ (bar) and CO_2_ flow rate, *X*_*Q*_CO_2___ (g/min).

The contour plots corresponding to the surfaces defined by Equation (8) for *Y*_DV_ and *Y*_SAF_, as functions of pressure, *X*_P_, and CO_2_ flow rate, *XQ*_CO__2_, are represented in Figure 1a,b, respectively. According to Figure 1a *Y*_DV_ increases with pressure for a given CO_2_ flow rate, except when CO_2_ flow rate is between 35 and 38 g/min, in which case *Y*_DV_ increases with increasing pressure, then decreases. This effect (excluding the exception interval) becomes more marked at lower flow rates. In general, *Y*_DV_ increases as CO_2_ flow rate diminishes for a fixed pressure. For the ranges studied, the highest *Y*_DV_ is found for high pressure (between 130 and 160 bar) and low CO_2_ flow rate (between 10 and 14 g/min). It is possible that greater *Y*_DV_ could be found with further increase of pressure and decrease of CO_2_ flow rate. On the other hand, as seen in Figure 1b, *Y*_SAF_ increases as CO_2_ flow rate decreases for a fixed value of pressure whereas, for a given value of CO_2_ flow rate, in general, *Y*_SAF_ first increases, then decreases for increasing pressure values. As a result, maximum values of *Y*_SAF_ occur for quite similar conditions to those of *Y*_DV_, that is, at high values of pressure (109–155 bar) and the lowest values of CO_2_ flow rate (between 10 and 20 g/min).

### 2.4. Enrichment Ratios Statistical Analysis

Enrichment parameters, *E*_i/j_ and relative enrichment parameters, *RE*_i_, are gathered in Table 1. The chromatographic analysis revealed that rosmarinic acid (RA) is completely retained in PV and therefore neither *E_RA/DV_* nor *RE*_RA_ are included in the results. For chlorogenic acid (CHA) and caffeic acid (CAF), *E*_i/PV_ values are significantly higher than *E*_i/DV_ which means that these two compounds are more concentrated in the PV than in the DV fraction.

Values of *RE*_CHA_ show that concentration of the CHA in PV was from 2.53 to 5.19 higher than concentration in DV. The major value was for run 11 (148 bar-17 g/min) whereas the lowest value was for run 1 (80 bar-35 g/min). On the other hand, values of *RE*_CAF_ show that the concentration of CAF in PV was from 1.14 to 8.17 higher than concentration in DV. The first value was for run 3 (92 bar-53 g/min) and the second one was for run 4 (120 bar-10 g/min). In this case, high values of *RE*_CAF_ are obtained with intermediate pressures and high CO_2_ flow rates.

In Table 2 the fitting coefficients of Equation (8) for *E*_CHA/PV_, *E*_RA/PV_ and *RE*_CHA_ are gathered. The remaining enrichment ratios could not be adjusted by the mathematical model. *E*_CHA/PV_ depends on all terms but only pressure (*β*_1_) was statistically significant. *E*_RA/PV_ do not depend on any of the quadratic terms and only pressure (*β*_1_) was statistically significant. *RE*_CHA_ depends on all terms, being statistically significant the pressure (*β*_1_) and the cross term (*β*_12_).

**Figure 2:**
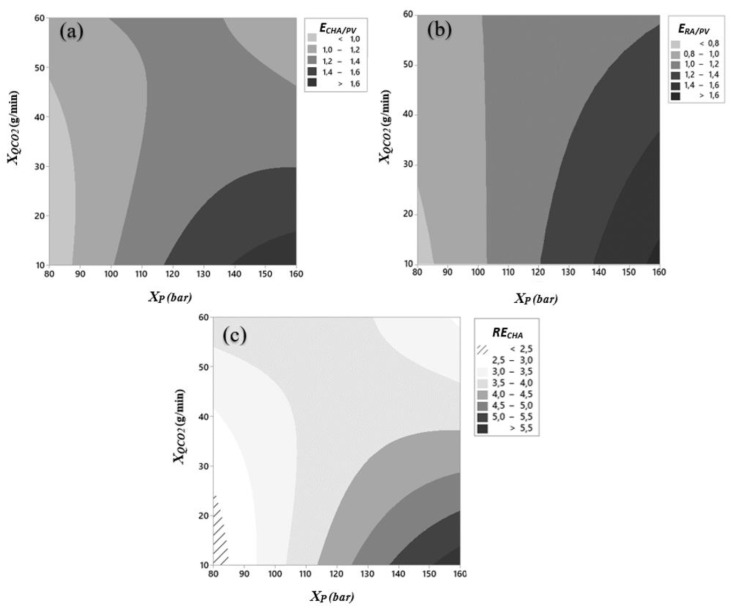
Contour plots of of the enrichment ratios: (**a**) *E*_CHA/PV_; (**b**) *E*_RA/PV_ and (**c**) *RE*_CHA_ as a function of pressure, *X*_P_ (bar) and CO_2_ flow rate, *XQ*_CO2_ (g/min).

The contour plots corresponding to the surfaces defined by Equation (8) for these enrichment ratios as functions of pressure, *X*_P_, and CO_2_ flow rate, *XQ*_CO2_, are depicted in Figure 2a–c. As can be observed in Figure 2a, at pressures higher than 116 bar, *E*_CHA/PV_ decreases as the flow rate increases. However, at lower values of pressure the flow rate barely has influence on the *E*_CHA/PV_. Regarding the influence of pressure, for CO_2_ flow rates below 46 g/min, the *E*_CHA/PV_ increases as the pressure increases, being the effect more pronounced as the lower is the CO_2_ flow rate. Between 46 and 60 g/min, for increasing values of pressure, *E*_CHA/PV_ increases, then decreases. The maximum was reached for high pressure values (138–160 bar) and low CO_2_ flow rate values (10–16 g/min).

In Figure 2b, the contour plot shows that below 120 bar the CO_2_ flow rate has not any influence on *E*_RA/PV_*,* except at lower pressure values where an increase in CO_2_ flow rate causes an increase in *E*_RA/PV_. From 120 bar onwards an increase in the flow leads to a decrease in *E*_RA/PV_. For a given CO_2_ flow rate, the higher the pressure, the higher *E*_RA/PV_. As occurred with *E*_CHA/PV_ the maximum *E*_RA/PV_ is placed at the highest values of pressure (155–160 bar) and the lowest values of CO_2_ flow rate (between 10 and 15 g/min).

Analysing Figure 2c it can be observed that up to 113 bar, when increasing CO_2_ flow rate, *RE*_CHA_ increases. However, at higher values of pressure, a decrease in is observed when CO_2_ flow rate increase. Referring to the influence of pressure, as it increases *RE*_CHA_ increases for CO_2_ flow rates below 47 g/min, being the effect more pronounced as the lower is the CO_2_ flow rate. The opposite takes place at CO_2_ flow rates above 54 g/min. Between 47 and 54 g/min, *RE*_CHA_ increases, then decreases. The maximum *RE*_CHA_ was reached for high pressure values (between 151 and 160 bar) and the lowest values of CO_2_ flow rate (10–14 g/min). Coupling the fact that CHA and CAF in a greater proportion in PV than in DV with the fact that rosmarinic acid only precipitates in PV, it can be concluded that the SAF technique can be used to obtain a dry solid highly enriched in antioxidant compounds. Taking into account the experimental inaccuracies, the statistical analysis for the applied RSM model provides the optimal working conditions to obtain the highest values of *E*_CHA/PV_, *E*_RA/PV_, and *RE*_CHA_ together with a maximum *Y_SAF_* are a pressure of 148 bar and a CO_2_ flow rate of 10 g/min.

The justification of the results obtained considering the different factors involved in the precipitation process is very complex, especially for these multicomponent systems [4]. Precipitation is a kinetic process governed by a driving force [52], that is expressed as the difference between the chemical potential of a supersaturated solution, *µ*, and the chemical potential in saturated solution *µ*_eq_. The sign of this difference indicates whether precipitation occurs (*µ* − *µ*_eq_ > 0) or if dissolution occurs instead (*µ* − *µ*_eq_ < 0). For the precipitation to occur it is necessary that the primary processes of nucleation and growth take place along with secondary processes of aggregation and rupture. It is in these primary and secondary processes that flow affects.

Focusing on the driving force of the precipitation phenomenon it is clear that solubility plays a determining role. Specifically, for the compounds of interest studied, the only experimental solubility data found in the literature in CO_2_ and in the scCO_2_-EtOH mixture are for caffeic acid [87,88] (Table S1 in Supplementary Materials). Taking into account the working conditions, the supersaturation in caffeic acid is guaranteed. With respect to chlorogenic and rosmarinic acids, the overstressing is even higher due to their higher concentration in the FS (Table 1) and because their solubilities are lower than those of caffeic acid as deduced from the Hildebrand solubility parameter [89], δ_H_, gathered in Table S1. The complete precipitation of RA in PV is a phenomenon that has been previously described in the literature [23,86]. This fact could be explained mainly by two effects, on the one hand, a lower solubility in the CO_2_-EtOH mixture, and on the other hand, the great supersaturation of the compound that favors the nucleation process and subsequent growth of the particles when compared to the two other compounds considered.

The optimal conditions reached for the maximum recovery of the fed material and the concentration of active ingredients (148 bar, 10 g/min) can be explained in a similar way. The flow rate value corresponds to the lower end of the studied interval, a fact that could be explained on the basis that this situation allows, on the one hand, a higher mass yield recovery due to a lower dragging effect, and on the other hand, a higher precipitation because nucleation is favored. Regarding pressure, it can be considered that, for a given flow, there are two factors that contribute in the opposite way to the driving force: nucleation and solubility. Both factors increase with pressure generating a maximum within the considered interval [90].

### 2.5. COSMO-RS as Screening Model for Antioxidants

Reliable Quantitative Structure Activity Relationship (QSAR) models can be very useful to predict activity of compounds from its structure, thus avoiding the, sometimes, laborious experimental measurements. In this case the possibility of attempting the development of a QSAR model for the antioxidant activity of compounds present in extracts of plants is explored.

**Figure 3:**
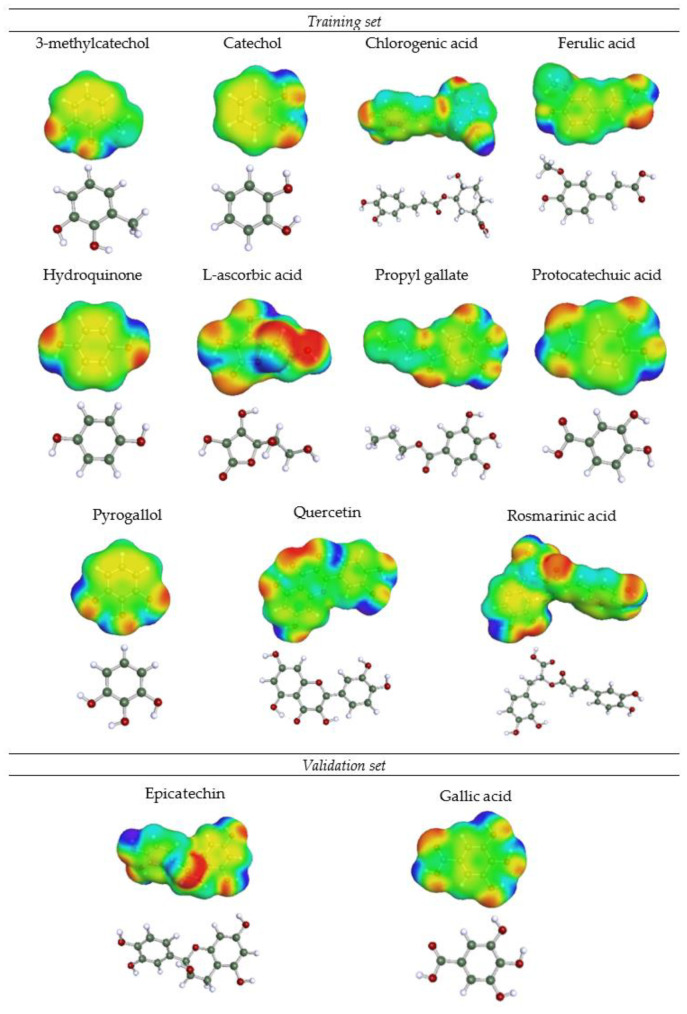
3D molecular structures of the compounds used in this work and their charge density (electronegative zone in red, electropositive zone in blue and neutral zone in green).

The model here developed is based on previous studies which used COSMO-RS on QSAR models [72,73,74]. Figure 3 shows the 3D structures of the compounds used in this work and their surface charge density in colours.

**Figure 4:**
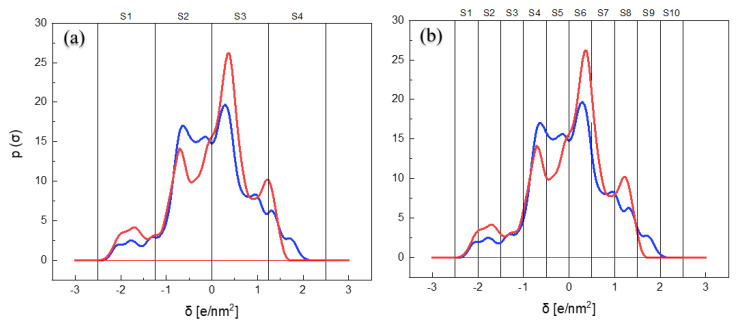
σ-profiles of rosmarinic acid (RA, red) and chlorogenic acid (CHA, blue) and its partition in (**a**) four intervals and (**b**) 10 intervals to generate the molecular descriptors.

To translate the σ-profile information into descriptors, that is, into numerical variables, σ-profile curves were divided in two different ways: a first thicker partition into four intervals [74,91] and a thinner partition into 10 different intervals [65,66,67,68], in order to be able to evaluate the suitability of the two models. Figure 4 shows an example of both divisions of the σ-profile for RA and CHA. The same division was applied to the σ-profiles of the rest of compounds. The descriptors of the model were defined as the area under the σ-profile curve for each interval i, S_i_, being these areas S_1_–S_4_ for the model with 4 partitions and S_1_–S_10_ for the model with 10 partitions. Table 3 shows the values of the molecular descriptors used for each molecule and their representation.

The σ-profiles obtained by COSMO-RS provide physicochemical information of the molecular structure based on its functional groups [92]. The molecules used in this work are phenolic type compounds and show characteristic peaks around σ = ±0.5 corresponding to the aromatic rings and which peaks that may be displaced due to the presence of other groups in the molecule. In addition, there are peaks in the hydrogen bond donor (HBD) region of −2.5 < σ < −1.0, that are characteristic of the hydrogen’s atoms in OH bonds, and some peaks in the hydrogen bond acceptor (HBA) region 1.0 < σ < 2.5, characteristic of hydrogen acceptor atoms such as oxygen atoms.

The antioxidant activity data, expressed as the value of *EC*_50_ (µM) for the DPPH assay of several compounds, were collected from the literature [93]. In this respect, it must be said that there are several chemical methods for determining antioxidant activity and not all of them measure the same activity because different antioxidant reaction mechanisms are involved in the different methods [93,94]. In this case, the DPPH assay has been chosen because it is widely used. *EC*_50_, which is the effective concentration of antioxidant compound that reduces 50% of the DPPH initially present in the assay, was transformed from (µM) to (mol substance/mol DPPH) in order to work with standardized values.

In this work Multiple Linear Regression (MLR) was used to establish the linear relationship between the molecular descriptors of the compounds and their antioxidant activity, represented as −*log*(*EC*_50_). Equation (9) provides the expression for MLR

(9) $$-log\left(EC_{50}\right)=a_{0}+a_{1}S_{1}+a_{2}S_{2}+a_{3}S_{3}+\ldots +a_{n}S_{n}$$

where *a*_0_, *a*_1_, *a*_2_, …, *a*_n_ are the regression coefficients and *S*_1_, *S*_2_, *S*_3_, …, *S*_n_ are the molecular descriptors. *Minitab^®^ 18* software was used to calculate the coefficients for Equation (9) as well as the associated statistical parameters.

In order to have a homogeneous database that would allow to assess the applicability of the QSAR model, 3 compounds were removed from the set of the 16 initial compounds chosen. Specifically: alpha-tocopherol for having a very different structure, trolox for not being a compound present in natural sources such as plants, and caffeic acid. Caffeic acid was subsequently eliminated because it has dimerization capacity and a preliminary QSAR model performed including it was not able to correctly adjust molecules with that dimerization capacity. Therefore, the remaining 13 compounds were divided by 80–20%: a subset of 11 compounds (training set) was built to carry out the MLR and the 2 remaining compounds were used as external validation. The compounds were selected to form the validation set according to the following criteria: (i) data of maximum and minimum antioxidant activity are excluded, (ii) data that are grouped in the upper or lower extreme of antioxidant activity are excluded and (iii) molecules that have the same functional group are excluded [95].

It should be noted that this number of compounds is too small to develop an effective QSAR model and only the possibility of developing a full model is explored. There are several reasons that hinder the use of a more extensive set. First, although there are many works on the antioxidant activity of extracts from plants, works on the antioxidant activity of pure compounds present in those extracts are surprisingly scarce. On the other hand, even for a given assay, such as DPPH, there are modifications of the method that not always provide the same results for a given compound. Finally, the antioxidant activity can be expressed in several ways and not all of them can be easily translated into each other because the lack of chemical information [93]. In this work, compounds were selected whose reported antioxidant activity has been considered reliable after a comparison with that provided by other sources.

Experimental values of *EC*_50_ (mol/mol) and the corresponding logarithms are gathered in Tables 5 and 6 for the compounds selected. Equations (10) and (11) describe the obtained linear relationship between the antioxidant activity and σ-profile descriptors for the 4 areas model and 10 areas model, respectively:

(10) $$-Log\left(EC_{50}\right)=-0.230+1.941S_{1}+0.519S_{2}-0.4055S_{3}-2.18S_{4}$$

The results show that, for the 4 areas model, all the descriptors (*S*_1_–*S*_4_) were statistically significant (*p* < 0.05) and the correlation coefficient, R^2^, was found to be 80.00%, indicating a good fitting of the model. The standard deviation was 0.17, the variation in the response explained by the model and adjusted for the number of predictors, R^2^(adjust), was 66.67% and the R^2^(pred), which determines how well the model would predict responses for new data, was 1.30%.

(11) $$-Log\left(EC_{50}\right)=-0.556-0.230S_{1}-0.317S_{4}+0.1903S_{5}-0.1015S_{6}+0.5683S_{7}$$

For the 10 areas model, out of the 10 initial descriptors, only *S*_4_–*S*_7_ were statistically significant (*p* < 0.05), although S_1_ is necessary to obtain a good correlation. R^2^ was found to be 93.14%, the standard deviation was 0.11, R^2^(adjust) was 86.28% and the R^2^(pred) was 72.68%. The fact that the *S*_4_–*S*_7_ areas significantly influence antioxidant activity can be explained on the basis of the reaction mechanism that the phenolic molecules considered in this work present in the DPPH method. This is a mechanism of transfer of a H atom to the radical according to which the antioxidant activity depends mainly on two factors: the H-abstraction level of the ground state molecule and the stability of the formed free radical [96]. It is in this last factor where the neutral zones of the molecule come in, since they are the zones that allow the delocalization of the free electron and therefore its stabilization. Although it is true that the σ-profile makes possible to identify the different areas of the molecules (presence of aromatic rings, alkyl groups, etc.) it does not seem to be precise enough to, for example, distinguish between different aromatic rings as is the case of RA [96]. Even so, it is precise enough to lead to an improvement when making more partitions over the previous model.

The antioxidant activity of compounds in the validation set was calculated from Equations (10) and (11) to test the performance of the models. Table 4 and Table 5 show the predicted values and the deviations from the experimental values as residues for *EC*_50_ (mol/mol) for each model. In the 4 areas model, both epicatechin and gallic acid values of EC_50_ are overestimated and present similar deviation values, −0.10 and −0.11, respectively. Although it appears that the model could predict the value of epicatechin, the deviation for gallic acid is greater than the experimental value itself. On one hand, in the 10 areas model, the epicatechin value of *EC*_50_ is underestimated and the residue is fairly low, 0.12, which means that the model could predict it quite correctly, while for gallic acid the value of *EC*_50_ is again overestimated almost in the same way as in the case of the 4 areas model being now the deviation −0.10.

The fact that the 10 areas model presents a better regression than the 4 areas model seems to indicate that a thinner partition of the σ-profile allows a greater correlation between the descriptors, based on the said profile, with the antioxidant activity. In addition, this partition has a chemical-physical sense behind it that allows, in a rough way, to relate each area to a part of the structure of the molecule. While in the 4-area model all partitions are significant, when the σ profile is divided into 10 segments, the model suggests that the most influential partitions are those related to the neutral regions of the molecules. As already seen, these areas take part in the delocalization and therefore in the stabilization of the free electron formed by abstracting an H. In any case, although the model fits well the data, it must be taken into account that the training set is really very small and the results provide only a clue that should be ascertained with a more extensive database.

## 3. Material and Methods

### 3.1. Plant Material

Dried sage leaves (*S. officinalis*) were purchased from a national supplier, Josenea Bio, in Pamplona (Spain). The plant was ground and sieved with a vibratory sieve shaker (*CISA* model BA 300N, Barcelona, Spain), and the particle size was adjusted to a normal distribution, being the average diameter approximately 0.5 mm. This average diameter was calculated according to ASAE S319.3 from the American National Standards Institute [97]. The moisture content of sage leaves, determined 10 times (*Sartorious* model MA 40 Moisture Analyzer, Goettingen, Germany) was 15.77 ± 0.96 wt.%.

### 3.2. Chemicals and Reagents

The solvents used in SFE, maceration and SAF were CO_2_ (Carburos Metálicos 99.9%, Zaragoza, Spain) and ethanol (VWR Chemicals, 99.9%, Barcelona, Spain). The HPLC-PDA mobile phase solvents were ethanol (PanReac AppliChem 99.9%, Barcelona, Spain), water (MilliQ 18.2 MΩ·cm, Zaragoza, Spain), formic acid (PanReac AppliChem 98%, Barcelona, Spain) and acetonitrile (Scharlau 99.9%, Barcelona, Spain). The HPLC-PDA standards used were rosmarinic acid (Sigma-Aldrich ≥98%, Madrid, Spain), caffeic acid (Sigma-Aldrich ≥98%, Madrid, Spain) and chlorogenic acid (European Pharmacopoeia Reference Standard 97.1%, Madrid, Spain).

### 3.3. Supercritical CO_2_ Extraction (SFE)

**Figure 5:**
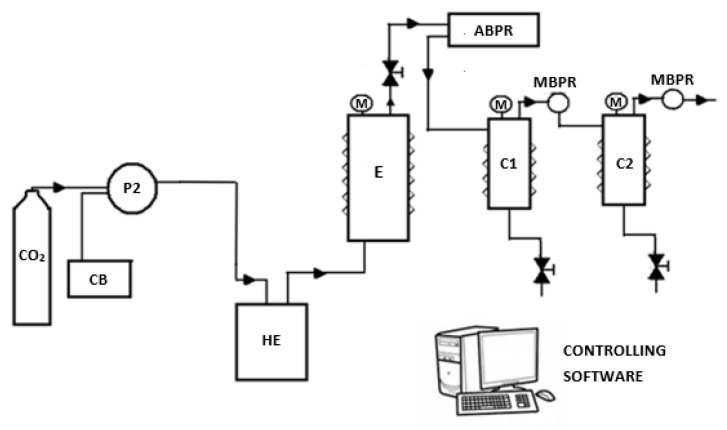
Scheme of the laboratory scale pilot plant. CO_2_ reservoir (R), cooling bath (CB), CO_2_ pump (P), heat exchanger (HE), extractor (E), collector 1 (C1), collector 2 (C2), automatic back pressure regulator (ABPR), manual back pressure regulator (MBPR), temperature and pressure gauges (M).

Defatting with sc-CO_2_ of the plant material was performed in a laboratory scale plant from Waters (model SFE-1000F-2-FMC10 System, PA, USA) whose scheme is represented in Figure 5. Its main parts are a 1 L extraction vessel or extractor (E) and two 0.5 L collectors (C1, C2), which are jacketed to be maintained at a constant temperature. CO_2_ from a bottle is kept liquid with a cooling bath (CB), and pumped by a pump (P2) through a heat exchanger (HE), that ensures it is above the critical temperature, into E. Temperatures, pressure in E and CO_2_ flow rate are automatically controlled. Pressure in the collectors is controlled by means of their respective manual back pressures (MBPR).

For each extraction, 100 g of plant material were loaded in the extractor along with 200 g of inert glass beads in order to obtain a better contact CO_2_-solid and facilitate the extraction process. CO_2_ was pumped until a pressure of 350 bar and a temperature of 40 °C were reached in E. Once the pressure and the temperatures were stable, the flow was stopped and a static stage (maceration) started. After 30 min, a dynamic stage of another 30 min began in which sc-CO_2_ was pumped with a flow of 60 g/min and passed through E to C1 and C2. Pressure and temperature were 90 bar and 45 °C in C1 and 30 bar and 30 °C in C2. The complete extraction process consisted of 4 static-dynamic cycles. The extracts were collected from the collectors after the total depressurization of the machine and the plant material was removed from the extractor and stored in a freezer until further maceration.

### 3.4. Maceration and Supercritical Antisolvent Fractionation (SAF) Processes

In total, 300 g of plant material, previously defatted with SFE, were macerated in 3 L absolute ethanol for 48 h at room temperature (25 °C). The solvent was removed with a rotatory evaporator (Büchi R-200, Flawil, Switzerland) to obtain the dry extract. This solvent-free extract was dissolved again in ethanol at 3% (wt.%) to prepare the feed solution (FS) for the SAF experiments. A laboratory scale plant was used to carry out the SAF experiments (Waters, PA, USA). The device was previously described [25], and its main components being a CO_2_ pump (P-SCF), a FS pump (P-LIQ), a 0.5 L precipitation vessel (PV) and a downstream vessel (DV). Pressure in PV, temperatures and flow rates of both CO_2_ and FS can be automatically controlled.

Several experimental parameters were fixed according to previous experience of the group: temperature in PV, studied on earlier works where it was seen that it has little relevance [24,25], was 40 °C to avoid thermal degradation of the compounds and FS flow rate was 0.45 mL/min. The FS concentration of 3% (wt.%) leads to a CO_2_ molar fraction which ensures the supercritical state of the mixture (CO_2_ + ethanol) at the operational conditions for all of the experiments [24]. The pressure in PV and the CO_2_ flow rate were varied (80–160 bar and 10–60 g/min, respectively).

A SAF experiment started by flowing supercritical CO_2_ through the plant. Once the selected conditions of pressure and temperature as well as of CO_2_ flow rate in PV and DV were stabilised, the FS, previously filtered through Cellulose Acetate 0.22 μm pore size filter, was pumped into the PV through an injector (nozzle Ø = 100 µm). Then, the insoluble compounds in the (CO_2_ + ethanol) mixture precipitate in PV while those compounds which remain soluble, were collected in DV. After FS has been completely passed through, 30 mL of pure ethanol were pumped to wash the remaining FS from the pipes, then only CO_2_ is pumped to remove ethanol from the solid precipitated in PV.

### 3.5. HPLC Analysis

**Figure 6:**
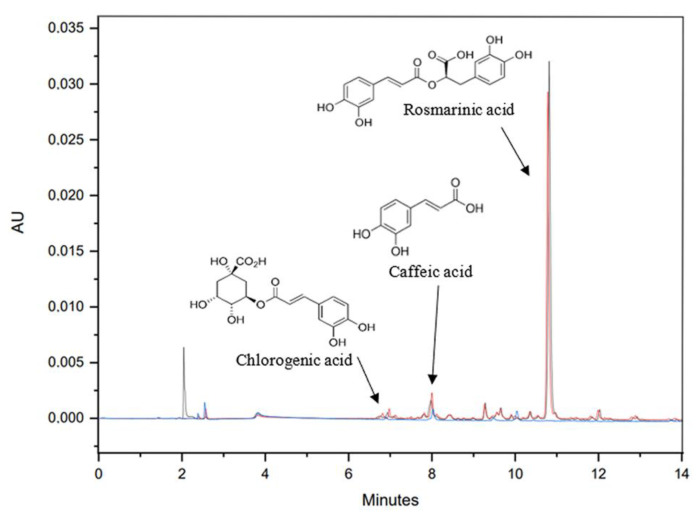
Chromatogram. Peak 1 CHA (T_R_ = 6.94 min, λ = 324 nm), peak 2 CAF (T_R_ = 7.98 min, λ = 324 nm) and peak 3 RA (T_R_ = 10.79 min, λ = 324 nm). PV fraction line red, DV fraction line black, FS fraction line blue.

Samples of FS as well as of PV and DV fractions were collected for their analysis with HPLC-PDA on a HPLC (Waters^®^ Alliance 2695, Milford, MA, USA) with a PDA (Waters^®^ 2998, MA, USA) detector. A CORTECS^®^ C18 2.7 μm (4.6 × 150 mm) (Waters^®^, MA, USA) with a pre-column CORTECS^®^ Pre-column VanGuard C18 2.7 μm (2.1 × 5 mm) (Waters^®^, MA, USA) were used. A gradient with the following solvents (A) 0.1% formic acid in Milli-Q water, (B) Milli-Q water and (C) acetonitrile, was used for the separation of the compounds. The gradient elution applied was: 10% A, 90–40% B, 0–50% C (0–15 min); 10% A, 40–10% B, 50–80% C (15–20 min), 10% A, 10–90% B, 80–0% C (20–25 min) and 10% A, 90% B, 0% C (25–30 min). The flow rate was 0.8 mL/min, the temperature was 30 °C and the detection wavelength was fixed at 324 nm. A total of approximately 100 ppm of each sample solution was filtered through a GH Polypropylene membrane ACRODISC 13 mm pore size 0.2 μm filter (Waters^®^, MA, USA). CHA, CAF and RA standards were run under the same chromatographic conditions. Retention times for each compound were 6.94 min, 7.98 min and 10.79 min, respectively, as can be observed in Figure 6. The analyses were performed in triplicate.

### 3.6. Experimental Design and Statistical Analysis

*Minitab^®^ 18,* Coventry, UK software was used to carry out a response surface methodology (RMS) based on central composite design (CCD) and provided 13 random experiments with five replicates in the central conditions according to the range levels of the two independent variables selected, as shown in Table 6. *Minitab^®^ 18* was also used to determine the values of each coefficient, *β,* in the model, Equation (8), as well as the significance of each term in Equation (8) (a term is considered significant if *p* < 0.05) and the optimal conditions for the maximum overall recovery yield and maximum bioactive compound (RA, CAF and CHA) enrichment.

### 3.7. QSAR Calculations

The structural descriptors for the molecules of pure compounds derived from their 2D σ-profiles of the molecules are obtained through COSMO-RS. The pre-optimized three-dimensional chemical structures of the compounds were obtained from the PubChem database. Those structures were refined using Gaussian 9.0 version with a DFT parametrization bvp86/dga1 and then COSMO-RS was used to generate the σ-profiles of the compounds. That parametrization was selected because he complies with the severe constraints imposed by the subsequent use of COSMO-RS to obtain the σ-profiles [98]. Then, σ-profiles were divided in several intervals and descriptors were defined as the areas under the σ-profile curve for each interval. The relationship between descriptors and antioxidant activity is described through a Multiple Linear Regression carried out by the *Minitab^®^ 18* software.

## 4. Conclusions

In this work, *Salvia officinalis* leaves were defatted by means of CO_2_ supercritical fluid extraction (SFE), then macerated in ethanol, being the yields of these processes 4.9% and 10.9%, respectively. Then the influence of pressure, which was set between 80 and 160 bar, and CO_2_ flow rate, which was set between 10 and 60 g/min, was studied for CO_2_ supercritical antisolvent fractionation (SAF) in a series of experiments designed through a response surface methodology (RMS) based on central composite design (CCD). Temperature and feed solution flow rate were kept constant (40 °C and 0.45 mL/min, respectively).

Overall recovery yields up to 85.1% were reached, obtaining a significantly higher percentage in the precipitation vessel (53.0–64.8%) than in the downstream vessel (7.1–28.5%). Chlorogenic acid and caffeic acid were mostly retained in the precipitation vessel fraction and the rosmarinic acid precipitated exclusively in this fraction. Then, a fine powder was obtained in the precipitation vessel, a powder highly enriched in antioxidants and free of organic solvents, with potential applications in the cosmetic, food or pharmaceutical industries. Statistical analysis of data leads to the prediction that optimal overall yield and enrichments can be simultaneously reached working at the conditions of 148 bar of pressure and 10 g/min of CO_2_ flow rate (composite desirability = 1.000). In view of these results and taking into account the complexity of the process, it should be noted the importance of the phenomena of supersaturation and solubility that constitute the driving force of the precipitation process. These, together with other secondary processes (macro-, meso- and micro-mixings, nucleation processes, aggregation and breakage) are of great importance for a possible industrial scaling of the process.

With respect to the preliminary attempt at a proposed QSAR model for the antioxidant activity, based on the division of σ-profiles provided by COSMO-RS when comparing the 4 areas model with the 10 areas model, the second presents better statistical results. The significant areas (S_4_–S_7_) indicate that the neutral zones of the molecule influence the antioxidant activity because they allow delocalization and therefore stabilization of the free electron formed by abstracting an H atom. The 10 areas model could become a promising tool with which it would be possible to estimate the antioxidant activity of pure compounds. However, a training data set including much more compounds is needed to ascertain this possibility. This would require the determination of the antioxidant activity, now lacking, of many pure compounds present in vegetable extracts as well as the use for that purpose of standardized methods of measuring and ways of expressing the antioxidant activity.

In view of the results achieved, it can be concluded that QSAR-COSMO-RS model, the advanced separation technologies and the experimental design used have been efficient tools for a screening and sustainable concentration of extracts enriched in antioxidants of interest.

## Figures and Tables

**Table 1:** Operational conditions of pressure, *X*_P_, and CO_2_ flow rate *X*_*Q*_CO_2___, for every run of the experimental design of the SAF process applied to the ethanolic extract from defatted *S. officinalis* leaves along with the corresponding results for the yields and enrichment ratios as defined by Equations (3)–(7).

Run	Exp. Run Order	*X*_P_ (bar)	*X*_*Q*_CO_2___ (g/min)	*Y*_PV_ (wt.%)	*Y*_DV_ (wt.%)	*Y*_SAF_ (wt.%)	*E*_CHA/PV_	*E*_CAF/PV_	*E*_RA/PV_	*E*_CHA/DV_	*E*_CAF/DV_	*RE*_CHA_	*RE*_CAF_
1	8	80	35	61.9	7.1	69.0	0.81	0.57	0.77	0.32	0.31	2.53	1.84
2	5	92	17	64.8	17.5	82.3	1.18	0.75	0.91	0.37	0.34	3.19	2.21
3	1		53	53.0	12.9	65.9	1.20	0.98	1.05	0.32	0.12	3.75	8.17
4	4	120	10	56.0	27.8	82.4	1.33	0.75	1.15	0.33	0.66	4.03	1.14
5	2		35	62.8	15.4	78.3	1.19	0.94	1.06	0.33	0.25	3.61	3.76
6	6		35	64.8	17.4	82.2	1.21	0.95	1.11	0.34	0.23	3.56	4.13
7	11		35	62.6	22.5	85.1	1.19	1.11	1.14	0.32	0.21	3.72	5.29
8	12		35	60.0	20.8	80.8	1.24	0.91	1.11	0.36	0.45	3.44	2.02
9	13		35	61.7	19.9	81.6	1.47	1.17	1.29	0.33	0.26	4.45	4.50
10	3		60	50.5	16.3	75.8	1.20	0.79	0.90	0.34	0.25	3.53	3.16
11	10	148	17	55.8	28.5	84.3	1.61	1.04	1.51	0.31	0.47	5.19	2.21
12	7		53	57.2	17.8	75.0	1.19	1.09	1.32	0.34	0.25	3.50	4.36
13	9	160	35	57.4	20.6	78.0	1.32	1.04	1.32	0.33	0.53	4.00	1.96

**Table 2:** Fitting coefficients of Equation (8) for DV and overall yields (*Y*_DV_ and *Y*_SAF_, respectively), chlorogenic and rosmarinic acid enrichment ratio in PV (*E*_CHA/PV_, *E*_RA/PV_, respectively) and chlorogenic acid relative enrichment ratio (*RE*_CHA_) along with the corresponding factors of significance of the terms, *p*. Regression coefficients, R^2^, and standard deviation, s, are also listed for each fitting.

	*Y*_DV_ */wt%*		*Y*_SAF_ */wt%*		*E*_CHA/PV_		*E*_RA/PV_		*RE*_CHA_	
	Coefficient Value	*p*	Coefficient Value	*p*	Coefficient Value	*p*	Coefficient Value	*p*	Coefficient Value	*p*
***β*** **_0_**	−40.1	0.000	8.8	0.000	−1.17	0.000	−0.341	0.000	−4.84	0.000
***β*** **_1_**	0.982	0.001	1.239	0.019	0.0326	0.013	0.01305	0.000	0.1019	0.008
***β*** **_2_**	−0.242	0.002	−0.2465	0.002	0.0141	0.136	0.0168	0.210	0.0964	0.122
***β*** **_11_**	−0.003	0.026	−0.00472	0.008	−0.000083	0.200	-	-	−0.000191	0.315
***β*** **_22_**	0.00549	0.082	-	-	0.000114	0.466	-	-	0.000353	0.455
***β_1_*** **_2_**	0.00303	0.217	-	-	−0.000218	0.117	−0.000164	0.150	−0.001116	0.019
**R^2^**	90.95	-	80.58	-	73.85	-	79.61	-	79.84	-
**s**	2.2	-	2.9	-	0.12	-	0.10	-	0.37	-

**Table 3:** Molecular descriptors for the 4 areas model and 10 areas model and their representations based on Lemaoui et al. representation [68].

4 Areas Model		10 Areas Model		Representation
Molecular Descriptor	Screening Charge Density Range (e/nm)	Molecular Descriptor	Screening Charge Density Range (e/nm)	
S_1_	−2.5 < σ < –1.25	S_1_	−2.5 < σ < –2.0	HBD region
		S_2_	2.0 < σ < –1.5	
		S_3_	−1.5 < σ < –1.0	
S_2_	−1.25 < σ < –0.0			
		S_4_	−1.0 < σ < –0.5	Non polar region with negative charges density
		S_5_	−0.5 < σ < 0.0	
S_3_	0.0 < σ < 1.25	S_6_	0.5 < σ < 1.0	Non polar region with positive charges density
		S_7_	0.0 < σ < 0.5	
		S_8_	1.0 < σ < 1.5	HBA region
S_4_	1.25 < σ < 2.5			
		S_9_	1.5 < σ < 2.0	
		S_10_	2.0 < σ < 2.5	

**Table 4:** Experimental and predicted values of *EC*_50_ and their logarithms for each compound used in QSAR model with 4 areas.

Compound	*EC*_50_ (mol/mol)	−*LogEC*_50_	*EC*_50_ (mol/mol)	−*LogEC*_50_	Deviation
	Experimental		Predicted		
Training set					
3-methylcatechol	0.29	0.54	0.22	0.65	0.07
Catechol	0.21	0.68	0.30	0.52	−0.09
Chlorogenic acid	0.39	0.41	0.55	0.26	−0.16
Ferulic acid	0.93	0.03	0.60	0.22	0.33
Hydroquinone	0.52	0.29	0.63	0.20	−0.11
L-ascorbic acid	0.21	0.68	0.19	0.71	0.02
Propyl gallate	0.08	1.08	0.08	1.09	0.00
Protocatechuic acid	0.62	0.21	0.62	0.21	0.00
Pyrogallol	0.17	0.78	0.25	0.60	−0.08
Quercetin	0.29	0.53	0.23	0.63	0.06
Rosmarinic acid	0.18	0.74	0.18	0.75	0.00
Validation set					
Epicatechin	0.22	0.67	0.32	0.50	−0.10
Gallic acid	0.10	0.98	0.21	0.67	−0.11

**Table 5:** Experimental and predicted values of *EC*_50_ and their logarithms for each compound used in QSAR model with 10 areas.

Compound	*EC*_50_ (mol/mol)	−*LogEC*_50_	*EC*_50_ (mol/mol)	−*LogEC*_50_	Deviation
	Experimental		Predicted		
Training set					
3-methylcatechol	0.29	0.54	0.24	0.62	0.05
Catechol	0.21	0.68	0.25	0.61	−0.04
Chlorogenic acid	0.39	0.41	0.39	0.41	0.00
Ferulic acid	0.93	0.03	0.93	0.03	−0.01
Hydroquinone	0.52	0.29	0.39	0.41	0.13
L-ascorbic acid	0.21	0.68	0.24	0.62	−0.03
Propyl gallate	0.08	1.08	0.08	1.09	0.00
Protocatechuic acid	0.62	0.21	0.51	0.29	0.10
Pyrogallol	0.17	0.78	0.22	0.66	−0.05
Quercetin	0.29	0.53	0.30	0.52	−0.01
Rosmarinic acid	0.18	0.74	0.18	0.75	0.00
Validation set					
Epicatechin	0.22	0.67	0.10	1.01	0.12
Gallic acid	0.10	0.98	0.20	0.7	−0.10

**Table 6:** Codification and levels of the two independent variables for the factorial design of SAF experiments.

Variable	Symbol	Factor Levels				
		{−1.44	−1	0	1	1.44}
**Pressure (bar)**	*X*_P_	80	92	120	148	160
**CO_2_ flow rate (g/min)**	*X*_Q_CO_2___	10	17	35	53	60

## Data Availability

Not applicable.

## Supplementary Materials

The following are available online at https://www.mdpi.com/article/10.3390/ijms22179351/s1.
