# Supplementary material for: Supercritical Antisolvent Fractionation of Antioxidant Compounds from Salvia officinalis
Source: Int J Mol Sci. 2021 Aug 28;22(17):9351. doi: 10.3390/ijms22179351 (PMC8431610; doi:10.3390/ijms22179351)
Supplement: Supplementary file 1 [file ijms-22-09351-s001.zip › Supplementary material.pdf]

Table S1. Hildebrand solubility parameter (δ_H) for the targeted compounds, EtOH and CO_2, and solubilities as mole fractions, x, in sc-CO_2 and sc-(CO_2 + EtOH) for the targeted compounds.

	δ_H	Solubility in sc-CO_2[c] 10^7·x	Solubility in sc-(CO_2 + ethanol)[c] 10^7·x
Caffeic acid	27.3[a]	0.008[d] (150 bar)<br>0.020[d] (200 bar)	5.8[e](200 bar; y_{EtOH} = 0.0223)
Chlorogenic acid	31.8[a]	-	-
Rosmarinic acid	33.8[a]	-	-
EtOH	25.7[a]	-	-
CO_2	6.8 – 17.8[b]	-	-

a Calculated by COSMOquick program for the QSRP approximation
b Reference 96
c at 313.15 K and atmospheric pressure
d Reference 94
f Reference 95

Cartesian Coordinates for Optimized Geometries in Gas Phase at bvp86

3-methylcatechol

O	0.04471200	-2.02969400	-0.00000100
O	-2.34237000	-0.91639900	-0.00000300
C	1.19623600	0.13027500	0.00000200
C	0.03706800	-0.65014100	0.00000000
C	1.01279700	1.52012000	-0.00000200
C	-1.25721000	-0.09452100	-0.00000500
C	2.55164900	-0.52434300	0.00001100
C	-0.24860300	2.11051000	-0.00000900
C	-1.38257500	1.29246400	-0.00001000
H	1.89577200	2.15542000	-0.00000200
H	2.70325300	-1.15027900	0.88735500
H	2.70317300	-1.15046900	-0.88721200
H	3.34059400	0.22982000	-0.00010500
H	-0.35405600	3.18931400	-0.00001400
H	-2.38066300	1.72239700	-0.00001300
H	0.97775800	-2.31403900	0.00007600
H	-1.96073200	-1.81960600	0.00002500

Protocatechuic acid

O	-2.22847200	1.82815100	-0.00004600
O	-3.18842000	-0.62053700	-0.00036300
O	3.13221000	-0.91018900	0.00016000
O	2.84494400	1.34759600	0.00034800
C	0.92976000	-0.07820900	0.00010800
C	0.02867800	0.99908700	0.00010900
C	-1.34341800	0.79782100	-0.00004900
C	0.41941800	-1.37813600	-0.00006300
C	-1.81864800	-0.53561000	-0.00021400
C	-0.95399600	-1.62728700	-0.00022200
C	2.37399600	0.22619200	0.00029200
H	0.42690900	2.01118200	0.00023900
H	1.10656500	-2.21905100	-0.00006800
H	-1.34246000	-2.64161600	-0.00034900
H	-3.10917400	1.39710200	-0.00016500
H	-3.42950900	-1.56531100	-0.00044300
H	4.05083700	-0.56562100	0.00021700

Propyl gallate

O	-2.04072200	0.10929400	0.00091600
O	2.22732000	2.68777500	0.00063200
O	3.68985800	-1.79561400	-0.00103800
O	4.19930900	0.82798200	-0.00051200
O	-1.45724700	-2.09942900	0.00073200
C	-4.30334300	0.89225300	-0.00196800
C	-3.41914200	-0.34231100	0.00148100
C	0.24531100	-0.41498500	0.00041800
C	1.26294800	-1.38364600	-0.00015600
C	0.56543200	0.94563400	0.00066500
C	-1.14151400	-0.92470900	0.00076800
C	1.89268300	1.36961900	0.00037300
C	2.58949900	-0.97247900	-0.00048200
C	-5.79075800	0.52562000	-0.00102700
C	2.90149700	0.39338800	-0.00021500
H	-4.06658500	1.50469900	0.87384800
H	-4.06649200	1.49980900	-0.88116000
H	-3.59688100	-0.96329900	0.88627400
H	-3.59615400	-0.96780000	-0.88026300
H	0.98628300	-2.43513300	-0.00032300
H	-0.22446500	1.68923400	0.00111900

Pyrogallol

O	0.06042100	-2.05757000	-0.00004700
O	-2.30387400	-0.79562400	0.00011600
O	2.39475800	-0.69594900	-0.00015900
C	0.01531400	-0.68575100	-0.00001500
C	-1.19144200	0.01767900	0.00007200
C	1.22897100	0.01235400	-0.00007000
C	-1.21164500	1.41399700	0.00010700
C	1.23961200	1.41220400	-0.00002800
C	0.01631700	2.08136700	0.00005800
H	-2.15021200	1.95846900	0.00017500
H	2.18458300	1.94288000	-0.00006900
H	0.01735400	3.16775100	0.00008800
H	-0.87721000	-2.33849000	-0.00010300
H	-3.08485400	-0.21302200	0.00008400
H	2.11714200	-1.63554300	-0.00020400

Ferulic acid

O	-2.59988000	1.65394300	-0.42433300
O	-3.92502100	-0.77927000	-0.19180700
O	4.67859400	1.41444200	-0.09957800
O	4.56793600	-0.84445400	0.16655500
C	0.27087200	-0.64324100	0.00383700
C	-1.91634600	0.48572200	-0.18700100
C	-0.53212700	0.51038200	-0.13815600
C	-0.42203200	-1.86119400	0.11313400
C	-2.56392400	-0.77353200	-0.09726500
C	-1.80975700	-1.93891900	0.06886300
C	1.71514800	-0.60651900	0.04653200
C	2.55634400	0.44079700	-0.04981100
C	-3.60534000	1.99494400	0.55539000
C	4.00998600	0.22821800	0.02118000
H	-0.06001000	1.48827000	-0.21779500
H	0.14826200	-2.78044200	0.23256400
H	-2.31259200	-2.90002100	0.14295700
H	2.19904100	-1.57887200	0.17648900
H	2.19840200	1.45970300	-0.18329600
H	-3.13827300	2.24081200	1.51500300
H	-4.32552200	1.18292700	0.69079500
H	-4.11335000	2.87722900	0.16345600
H	-4.20638900	-1.71118800	-0.10804700
H	5.62046600	1.14433900	-0.03903100

Catechol

O	-1.68804500	-1.41784000	-0.00000100
O	-1.79000100	1.21031500	0.00001400
C	-0.50992000	-0.73752700	-0.00000200
C	-0.52147200	0.67401200	0.00000500
C	0.71960100	-1.39029500	-0.00001000
C	0.65820800	1.40817600	0.00000500
C	1.91738000	-0.66675500	-0.00001100
C	1.88118700	0.72536900	-0.00000400
H	0.72869800	-2.47680900	-0.00001600
H	0.62671300	2.49448000	0.00001000
H	2.86460700	-1.19440900	-0.00001800
H	2.80551000	1.29415100	-0.00000400
H	-2.37253000	-0.71554100	0.00000600
H	-1.69852400	2.18045400	0.00002100

Hydroquinone

O	2.77546000	0.11009100	-0.00003100
O	-2.77546700	-0.11008400	0.00004300
C	1.40981100	0.02905700	-0.00001700
C	-1.40981200	-0.02906300	0.00001600
C	0.67834400	1.22230800	-0.00003000
C	-0.71410400	1.18596600	-0.00001000
C	0.71410900	-1.18597400	0.00001100
C	-0.67834400	-1.22230500	0.00002500
H	1.20532200	2.17108800	-0.00005300
H	-1.26703600	2.12310400	-0.00001600
H	1.26704000	-2.12311200	0.00001900
H	-1.20531300	-2.17109000	0.00004300
H	3.10203200	-0.80925800	-0.00004300
H	-3.10201600	0.80927400	-0.00001200

L-ascorbic acid

O	-0.17776900	-1.17794100	-0.59067500
O	1.24313500	0.32578800	1.43394500
O	-0.82944100	2.40393700	-0.31305900
O	3.81846800	-0.18321300	0.27389900
O	-3.16346700	0.50016700	0.48292300
O	-2.14644900	-2.12401400	0.12661100
C	0.26134900	0.19005900	-0.78060200
C	1.52900800	0.41240700	0.05766300
C	-0.88756900	1.05555500	-0.32297900
C	2.63239600	-0.53382100	-0.41236900
C	-1.91553200	0.25183500	0.02436100
C	-1.49163700	-1.13486400	-0.12498300
H	0.47205400	0.32806100	-1.85087000
H	1.88036600	1.44266900	-0.11441100
H	2.30475300	-1.56668300	-0.20939200
H	2.73343400	-0.43281800	-1.50695900
H	0.82199800	-0.55120600	1.54386100
H	0.10997500	2.64305000	-0.44222000
H	4.47596000	-0.85646400	0.02119900
H	-3.52243500	-0.41143400	0.60309200

Gallic acid

O	-3.06967700	-0.09278800	0.00000400
O	-1.86069800	2.28832200	-0.00000200
O	-1.68506900	-2.42321900	0.00001000
O	3.14119400	-1.10765800	-0.00007000
O	3.16366800	1.16795900	-0.00004400
C	1.07103300	0.01656600	0.00002900
C	-1.70283900	-0.03222400	0.00000900
C	-1.02768300	1.19580500	0.00001100
C	-1.00122900	-1.24803400	0.00001000
C	0.36066000	1.22796400	0.00002100
C	0.39194200	-1.20511300	0.00002000
C	2.54361100	0.12010500	0.00004900
H	0.91729900	2.16173900	0.00002700
H	0.94710900	-2.13757200	0.00002300
H	-3.36694100	0.84077200	-0.00001300
H	-1.29392700	3.08164800	0.00002100
H	-2.63031100	-2.16524000	0.00000200
H	4.09845300	-0.89268800	-0.00014200

Epicatechin

O	0.45540000	0.75710000	-0.11140000
O	-0.01980000	-1.31810000	1.82830000
O	4.33340000	-2.14530000	-0.00430000
O	4.82150000	2.57760000	-0.31710000
O	-5.11890000	-0.82250000	-1.52540000
O	-5.80480000	0.96340000	0.49110000
C	0.05280000	-1.61210000	0.43270000
C	-0.37070000	-0.38570000	-0.38650000
C	1.48800000	-1.99160000	0.07350000
C	2.35960000	-0.76960000	-0.03670000
C	1.80440000	0.51290000	-0.12990000
C	-1.82670000	-0.02720000	-0.15390000
C	3.75340000	-0.91210000	-0.06740000
C	2.63180000	1.63610000	-0.21070000
C	-2.81880000	-0.59310000	-0.95290000
C	-2.16940000	0.86830000	0.85810000
C	4.57790000	0.20960000	-0.16230000
C	4.01720000	1.48310000	-0.22970000
C	-4.15710000	-0.26280000	-0.73960000
C	-3.50760000	1.19870000	1.07130000
C	-4.50140000	0.63310000	0.27260000
H	-0.60300000	-2.47070000	0.24560000
H	-0.25540000	-0.60340000	-1.45860000
H	1.50070000	-2.50790000	-0.89400000
H	1.88790000	-2.66640000	0.83950000
H	2.18970000	2.62820000	-0.26570000
H	-2.54910000	-1.29320000	-1.73990000
H	-0.95900000	-1.29610000	2.07780000
H	-1.41380000	1.31490000	1.49840000
H	5.65940000	0.09890000	-0.18250000
H	-3.76220000	1.89650000	1.86460000
H	5.30020000	-2.04600000	-0.03830000
H	4.26610000	3.37520000	-0.35530000
H	-4.69620000	-1.41580000	-2.17000000
H	-5.85210000	1.59450000	1.22970000

Quercetin

O	-0.26244800	-0.70235200	-0.14354000
O	0.38662800	2.88854000	0.27534600
O	-4.48931000	1.52013100	0.17369200
O	-2.33558500	2.83674700	0.26067200
O	-4.21238300	-3.23188400	-0.24740000
O	4.41422600	-2.29224700	0.67480800
O	5.99482700	-0.35883100	-0.16416900
C	-2.35096500	0.47940400	0.01906500
C	-1.64065000	-0.72524100	-0.10055900
C	0.41274000	0.50386100	-0.06774000
C	1.85361000	0.29921800	-0.11097100
C	-0.24697800	1.69656000	0.10112900
C	-1.70028400	1.75743900	0.13215900
C	-3.78161600	0.39778500	0.05302800
C	-2.23151200	-1.97996900	-0.19129800
C	-3.63362700	-2.00353100	-0.15642400
C	2.41508400	-0.93039800	0.29899300
C	-4.41450000	-0.84398800	-0.03585800
C	2.75281600	1.28036300	-0.57255700
C	3.78122900	-1.14236800	0.28474700
C	4.13434800	1.08926400	-0.59889600
C	4.65835500	-0.12587000	-0.16079600
H	-1.64997400	-2.88695500	-0.29101200
H	1.75598200	-1.72091300	0.65195200
H	-5.49906300	-0.89921500	-0.01068400
H	2.36771000	2.20389600	-1.00157700
H	4.80287200	1.85820300	-0.97075400
H	1.32495400	2.67522100	0.47692300
H	-3.76189300	2.26303000	0.22938600
H	-5.17763000	-3.08648500	-0.21230100
H	3.72946600	-2.92780900	0.95518600
H	6.09164300	-1.27497200	0.17349800

Rosmarinic acid

O	-1.19801500	1.09459800	-0.39318100
O	-3.07306300	4.11522700	0.13639900
O	-2.61589600	-3.55425700	-0.66426400
O	-4.74139600	-4.02555600	0.82813600
O	-1.18923100	3.74733200	-1.08537800
O	-0.07163400	2.22143800	1.24738700
O	7.01278400	0.07562400	1.40305200
O	7.26010900	-1.66704000	-0.56933300
C	-3.55845200	1.33253400	-0.73955100
C	-2.33029200	1.88253200	-0.00139000
C	-3.89766300	-0.07255300	-0.31946900
C	-3.09747400	-1.16646400	-0.68798600
C	-5.01179100	-0.35549200	0.47127100
C	-2.08535400	3.34028100	-0.38615300
C	-3.37452800	-2.47692800	-0.31439300
C	-5.32437100	-1.65803500	0.87227100
C	-4.51296100	-2.72278700	0.48604900
C	-0.06563200	1.41570400	0.33757900
C	3.53506100	0.10613100	0.12965600
C	1.07360300	0.63458300	-0.15164300
C	2.28090600	0.75947000	0.43362600
C	4.68654500	0.38705400	0.89483800
C	3.68338200	-0.82463900	-0.91823300
C	5.91640000	-0.20800700	0.65343100
C	4.89812000	-1.44445200	-1.19585100
C	6.00672400	-1.13356100	-0.40910800
H	-4.39864300	1.99873900	-0.52262900
H	-3.36609100	1.38615400	-1.81752200
H	-2.47496900	1.82595700	1.08369900
H	-2.21781000	-0.98759700	-1.30493200
H	-5.66462900	0.45503600	0.78579100
H	-6.20132300	-1.84671500	1.48632900
H	0.90146000	-0.03251100	-0.99277300
H	2.31877300	1.46577400	1.26795600
H	4.61693500	1.10088100	1.71436100
H	2.82390900	-1.06989200	-1.53784300
H	4.98623000	-2.15758200	-2.01085000
H	-2.84756200	5.01800200	-0.17786400
H	-1.87865000	-3.19756300	-1.19604700
H	-5.55248000	-4.02439100	1.37136400
H	7.72567300	-0.47691100	1.01875400
H	7.22657300	-2.28853900	-1.31999700

Chlorogenic acid

O	0.75915900	-0.88140600	-0.27476600
O	4.29987400	0.81466400	1.35898200
O	2.32011700	-3.06830500	0.16949600
O	4.56225200	-1.84324700	1.44728400
O	4.72083900	2.09675300	-1.95490400
O	5.46278000	2.74142000	0.09395400
O	0.13667900	0.76762400	1.18983400
O	-7.30653000	1.53018300	0.99813900
O	-8.07913800	-0.27640100	-0.75779400
C	4.19167100	0.71494600	-0.06291300
C	2.14244900	-0.65658700	0.13801600
C	2.70466900	0.63408700	-0.44503100
C	4.95963900	-0.52459300	-0.58453300
C	2.88717400	-1.88796800	-0.35456400
C	4.36737900	-1.79625300	0.05040100
C	4.85293400	1.96559400	-0.62235100
C	-0.16252500	-0.08117000	0.37171000
C	-1.51074600	-0.41664900	-0.09985600
C	-2.58364100	0.24063300	0.38561000
C	-3.98516300	0.08481000	0.07485100
C	-4.95200600	0.89961900	0.70695800
C	-4.47716500	-0.85740400	-0.85134000
C	-6.30314000	0.78354900	0.43505000
C	-5.82718600	-1.00101700	-1.14788800
C	-6.75104300	-0.17436400	-0.50108800
H	2.16765100	-0.61568000	1.23356800
H	2.60416500	0.62967700	-1.53464000
H	2.16411500	1.49312600	-0.03827600
H	6.01775300	-0.43765700	-0.31764800
H	4.88864800	-0.56770600	-1.67473200
H	2.84830100	-1.88305700	-1.46173300
H	4.87241600	-2.67581300	-0.37439400
H	4.92909000	1.57131300	1.47081500
H	1.35775700	-2.90989700	0.08787500
H	4.53131100	-0.89789300	1.72980300
H	5.21099700	2.92033900	-2.17162900
H	-1.60316400	-1.20386900	-0.84454700
H	-2.35534600	1.01052900	1.12858900
H	-4.61911200	1.64374100	1.43126700
H	-3.77033300	-1.50789900	-1.36262000
H	-6.17910900	-1.73515500	-1.86467100
H	-6.89522500	2.15066500	1.62786300
H	-8.49797300	0.40156000	-0.18488000
